# Association Studies of the GPR103 and BCL2L15 Genes in Autoimmune Thyroid Disease in the Japanese Population

**DOI:** 10.3389/fendo.2016.00092

**Published:** 2016-07-19

**Authors:** Yoshiyuki Ban, Teruaki Tozaki, Yasuko Nakano

**Affiliations:** ^1^Third Department of Internal Medicine, Teikyo University Chiba Medical Center, Ichihara, Chiba, Japan; ^2^Department of Pharmacogenomics, Showa University School of Pharmacy, Tokyo, Japan

**Keywords:** Graves’ disease, Hashimoto’s thyroiditis, genetics, autoimmunity, association

## Abstract

While the past genome-wide association study (GWAS) for autoimmune thyroid diseases (AITDs) was done in Caucasians, a recent GWAS in Caucasian patients with both AITD and type 1 diabetes [a variant of autoimmune polyglandular syndrome type 3 (APS3v)] identified five non-*HLA* genes: *BCL2L15, MAGI3, PHTF1, PTPN22*, and *GPR103*. The aim of our study was to replicate these associations with AITD in a Japanese population. Since analyzing the rs2476601 single-nucleotide polymorphism (SNP) within the *PTPN22* gene revealed no polymorphism in the Japanese, we analyzed four SNPs, rs2358994 (in *BCL2L15*), rs2153977 (in *MAGI3*), rs1111695 (in *PHTF1*), and rs7679475 (in *GPR103*) genotypes in a case–control study based on 447 Japanese AITD patients [277 Graves’ disease (GD) and 170 Hashimoto’s thyroiditis (HT) patients] and 225 matched Japanese controls using the high-resolution melting and unlabeled probe methods. Case–control association studies were performed using the χ^2^ and Fisher’s exact tests with Yates correction. The G allele of rs7679475 (A/G) was associated with HT compared with controls [*P* = 0.022, odds ratio (OR) = 0.69]. GD showed no significant associations with any SNPs. However, when patients with GD were stratified according to Graves’ ophthalmopathy (GO), the G allele of rs2358994 (A/G) was associated with GO vs. controls (*P* = 0.018, OR = 1.52). These findings suggest that in the Japanese population the *GPR103* gene may contribute to the pathogenesis of HT. Moreover, this study demonstrated that the SNP rs2358994 within *BCL2L15* gene is associated with GO in the Japanese population.

## Introduction

Autoimmune thyroid diseases (AITDs), including Graves’ disease (GD) and Hashimoto’s thyroiditis (HT), are among the most common human autoimmune diseases. The prevalence in Caucasians is 1% (1, 2), and the prevalence in Japanese is not known. GD is characterized clinically by hyperthyroidism, diffuse goiter, and the presence of thyrotropin receptor (TSHR) antibodies. Some patients develop extrathyroidal manifestations, mainly ophthalmopathy and dermopathy [reviewed by Davies (3)]. Stimulatory TSHR autoantibodies are directly responsible for hyperthyroidism in GD. However, despite their contrasting clinical presentations, GD and HT share many common features, including the presence of autoantibodies to thyroid peroxidase and/or thyroglobulin, but rarely to the TSHR (3–5).

The pathogenesis of AITDs is thought to involve several risk factors, including genetic risk factors [reviewed in Ref. (6)] and environmental triggers, such as cigarette smoking, iodine intake, and infection (7, 8) [reviewed by Davies (3)]. However, the evidence for interactions between hereditary factors and environmental influences appears to be much stronger for cigarette smoking and iodine intake than for infections (8).

While the past genome-wide association study (GWAS) for AITDs was done in Caucasians, Lombardi et al. identified three new single-nucleotide polymorphisms (SNPs) on chromosome 3q that are associated with GD in Italian patients indicating potential GD susceptibility genes in this locus (9). Recently, Immunochip genetic association analysis identified 30 SNPs in several genes that were significantly associated with Young-Age-of-Onset GD, including major histocompatibility complex class I and class II genes, *BTNL2, NOTCH4, TNFAIP3*, and *CXCR4* (10).

Moreover, the first GWAS performed in patients that developed both type 1 diabetes (T1D) and AITD [a variant of autoimmune polyglandular syndrome type 3 (APS3v)] was reported (11). Indeed, the co-occurrence of T1D and AITD in the same individual is classified as a variant of the autoimmune APS3v. They identified multiple signals within the *HLA* region, and conditioning studies suggested that a few of them contributed independently to the strong association of the *HLA* locus with APS3v (11). Outside the HLA region, variants in G protein-coupled receptor 103 (*GPR103*) located on chromosome 4q27, a gene not suggested by previous studies of APS3v, T1D, or AITD, showed genome-wide significance (*P* < 5 × 10^−8^) (11). Moreover, a locus on 1p13 containing four genes [B-cell lymphoma 2-like protein 5 (*BCL2L15*), membrane-associated guanylate kinase, WW and PDZ domain-containing 3 (*MAGI3*), putative homeodomain transcription factor 1 (*PHTF1*), and protein tyrosine phosphatase non-receptor type 22 (*PTPN22*)] showed genome-wide significant associations (11). Because of possible genetic heterogeneity between different ethnic groups, we aimed to replicate in the Japanese population the associations between AITD and the four SNPs, rs2358994 (in *BCL2L15*), rs2153977 (in *MAGI3*), rs1111695 (in *PHTF1*), and rs7679475 (in *GPR103*), recently reported in Caucasians. Types of all these SNPs we studied are nonsense. Since analyzing the rs2476601 SNP within the *PTPN22* gene revealed no polymorphism in the Japanese, we did not analyze it in the present study.

## Materials and Methods

### Ethics Statement

The research protocol was approved by the Ethic Committee and each subject signed the informed consent form approved by the Institutional Review Board.

### Patients and Controls

#### AITD Patients

Four hundred forty-seven unrelated Japanese AITD patients (age range, 30–80 years) were studied. There were a total of 277 GD patients (69 males and 208 females) and 170 HT patients (17 males and 153 females). Of the 277 GD patients, 89 (32.1%) had Graves’ ophthalmopathy (GO).

#### Clinical Assessment

Graves’ disease was diagnosed based on clinical symptoms and biochemical confirmation of hyperthyroidism, including diffuse goiter, elevated radioactive iodine uptake, and elevated thyroid hormone levels. Ophthalmopathy was classified according to the system recommended by the American Thyroid Association (ATA) Committee (12). Eighty-nine of the GD patients showed ophthalmopathy defined as ATA class III or greater and were classified as GO. HT patients had documented clinical and biochemical hypothyroidism requiring thyroid hormone replacement therapy and showed autoantibodies against thyroid peroxidase with or without antibodies against thyroglobulin.

#### Controls

Two hundred twenty-five age- and sex-matched healthy Japanese volunteers (79 males and 146 females; age range, 34–72 years) served as controls in our association studies. All controls had no personal or family history of any autoimmune disease.

### SNP Typing

DNA was extracted from whole blood using the Puregene kit (Gentra Systems, Minneapolis, MN, USA). Four SNPs (rs2358994, rs2153977, rs1111695, and rs7679475) were genotyped by the high-resolution melting and unlabeled probe methods using LightScanner^®^ (Idaho Technology Inc., Salt Lake City, UT, USA) based on the manufacture’s protocol.

### Statistical Analysis

Case–control analysis and Hardy–Weinberg equilibrium (HWE) test of SNP were performed using SNPALyze ver. 7.0 (Dynacom, Yokohama, Japan) (13). Differences in the allele frequencies between the groups were analyzed using the chi-square test for two by two and two by three and Fisher’s exact test with Yates correction. The odds ratio (OR) was calculated using the modified method of Woolf (14). A *P*-value of <0.05 was considered statistically significant. HWE tests were carried out for all loci among subjects and controls separately.

## Results

Tests in subjects and controls did not show any significant deviation from HWE for any of the SNPs (data not shown). Analyzing the rs2476601 SNP within the *PTPN22* gene revealed no polymorphism in the Japanese (15). Table 1 shows the allele and genotype frequencies of four SNPs at the two risk loci (1p13 and 4q27) in AITD patients and controls. SNP rs7679475 (A/G) within the *GPR103* gene showed significant association with HT. The G allele of rs7679475 was present in 22.9% of HT patients and 30.2% of controls [*P* = 0.023, OR = 0.69, 95% confidence interval (CI): 0.4976–0.9495], and the distribution of genotype frequencies differed significantly between HT patients and controls (*P* = 0.046). There were no significant associations between HT and other three SNPs. GD showed no significant associations with any SNPs.

**Table 1:** **Case–control association results for the four SNPs at the two risk loci (1p13 and 4q27)**.

SNP	CHR	Gene	Allele/genotype	Control (*n* = 225)	AITD (*n* = 447)	*P* value^a^ (OR) AITD vs. control	GD (*n* = 277)	*P* value^a^ (OR) GD vs. control	HT (*n* = 170)	*P* value^a^ (OR) HT vs. control
rs2358994	1p13	BCL2L15	A	257 (57.1)	481 (53.8)		301 (54.3)		180 (52.9)	
			G	193 (42.9)	413 (46.2)	0.25	253 (45.7)	0.38	160 (47.1)	0.24
			A/A	73 (32.4)	130 (29.1)		84 (30.3)		46 (27.1)	
			A/G	111 (49.3)	221 (49.4)		131 (48.0)		88 (51.8)	
			G/G	41 (18.2)	96 (21.5)	0.51	60 (21.7)	0.62	36 (21.2)	0.48
rs2153977	1p13	MAGI3	T	269 (59.8)	506 (56.6)		319 (57.6)		187 (55.0)	
			C	181 (40.2)	388 (43.4)	0.27	235 (42.4)	0.48	153 (45.0)	0.18
			T/T	84 (37.3)	144 (32.2)		94 (33.9)		50 (29.4)	
			T/C	101 (44.9)	218 (48.8)		131 (47.3)		87 (51.2)	
			C/C	40 (17.8)	85 (19.0)	0.42	52 (18.8)	0.73	33 (19.4)	0.25
rs1111695	1p13	PHTF1	T	83 (18.4)	186 (20.8)		114 (20.6)		72 (21.2)	
			G	367 (81.6)	708 (79.2)	0.31	440 (79.4)	0.40	268 (78.8)	0.34
			T/T	12 (5.3)	27 (6.0)		17 (6.1)		10 (5.9)	
			T/G	59 (26.2)	132 (29.5)		80 (28.9)		52 (30.6)	
			G/G	154 (68.4)	288 (64.4)	0.59	180 (65.0)	0.72	108 (63.5)	0.59
rs7679475	4q27	GPR103	A	314 (69.8)	660 (73.8)		398 (71.8)		262 (77.1)	
			G	136 (30.2)	234 (26.2)	0.12	156 (28.2)	0.47	78 (22.9)	0.023 (0.69)^b^
			A/A	109 (48.4)	242 (54.1)		144 (52.0)		98 (57.6)	
			A/G	96 (42.7)	176 (39.4)		110 (39.7)		66 (38.8)	
			G/G	20 (8.9)	29 (6.5)	0.28	23 (8.3)	0.73	6 (3.5)	0.046^c^

*Values given are the number of subjects, with the percentage in parentheses*.

*SNP, single-nucleotide polymorphism; CHR, chromosome; OR, odds ratio*.

*^a^P-value based on χ^2^ distribution*.

*^b^The distribution of allele frequencies differed significantly between HT patients and controls*.

*^c^The distribution of genotype frequencies differed significantly between HT patients and controls*.

Since cell-mediated immunity plays a central role in the pathogenesis of GO, similar to Crohn’s disease and rheumatoid arthritis (16), we tested the four SNPs for association with the subset of our GD patients that had GO (Table 2). SNP rs2358994 (A/G) within the B-cell lymphoma 2-like protein 15 (*BCL2L15*) gene was associated with GO compared with controls (*P* = 0.018, OR = 1.52, 95% CI: 1.08–2.16) and GD without GO (*P* = 0.012, OR = 1.58, 95% CI: 1.10–2.27) (Table 2). The G allele of rs2358994 was present in 53.4% of GO patients and 42.9% of controls (*P* = 0.018, OR = 1.52, 95% CI: 1.08–2.16), and the distribution of genotype frequencies differed significantly between GO patients and controls (*P* = 0.033). There were no significant associations between GO and other three SNPs. These data suggested that the *BCL2L15* gene may be associated with GO.

**Table 2:** **Frequencies of alleles and genotypes of the four SNPs in Graves’ patients with and without ophthalmopathy (GO)**.

SNP	Allele/genotype	Control (*n* = 225)	GD (*n* = 277)	GO (*n* = 89)	GD without GO (*n* = 188)	*P* value^a^ (OR) GO vs. control	*P* value^a^ (OR) GO vs. GD without GO	*P* value^a^ (OR) GD without GO vs. control
rs2358994	A	257 (57.1)	301 (54.3)	83 (46.6)	218 (58.0)			
	G	193 (42.9)	253 (45.7)	95 (53.4)	158 (42.0)	0.018 (1.52)^b^	0.012 (1.58)^d^	0.80
	A/A	73 (32.4)	84 (30.3)	16 (18.0)	68 (36.2)			
	A/G	111 (49.3)	133 (48.0)	51 (57.3)	82 (43.6)			
	G/G	41 (18.2)	60 (21.7)	22 (24.7)	38 (20.2)	0.033^c^	0.0086^e^	0.51
rs2153977	T	269 (59.8)	319 (57.6)	94 (52.8)	225 (59.8)			
	C	181 (40.2)	235 (42.4)	84 (47.2)	151 (40.2)	0.11	0.12	0.99
	T/T	84 (37.3)	94 (33.9)	28 (31.5)	66 (35.1)			
	T/C	101 (44.9)	131 (47.3)	38 (42.7)	93 (49.5)			
	C/C	40 (17.8)	52 (18.8)	23 (25.8)	29 (15.4)	0.25	0.12	0.63
rs1111695	T	83 (18.4)	114 (20.6)	38 (21.3)	76 (20.2)			
	G	367 (81.6)	440 (79.4)	140 (78.7)	300 (79.8)	0.41	0.76	0.52
	T/T	12 (5.3)	17 (6.1)	8 (9.0)	9 (4.8)			
	T/G	59 (26.2)	80 (28.9)	22 (24.7)	58 (30.9)			
	G/G	154 (68.4)	180 (65.0)	59 (66.3)	121 (64.4)	0.49	0.28	0.58
rs7679475	A	314 (69.8)	398 (71.8)	122 (68.5)	276 (73.4)			
	G	136 (30.2)	156 (28.2)	56 (31.5)	100 (26.6)	0.76	0.23	0.25
	A/A	109 (48.4)	144 (52.0)	42 (47.2)	102 (54.3)			
	A/G	96 (42.7)	110 (39.7)	38 (42.7)	72 (38.3)			
	G/G	20 (8.9)	23 (8.3)	9 (10.1)	14 (7.4)	0.94	0.50	0.49

*Values given are the number of subjects, with the percentage in parentheses*.

*SNP, single nucleotide polymorphism; OR, odds ratio*.

*^a^P-value based on χ^2^ distribution*.

*^b^The distribution of allele frequencies differed significantly between GO patients and controls*.

*^c^The distribution of genotype frequencies differed significantly between GO patients and controls*.

*^d^The distribution of allele frequencies differed significantly between GD with and without GO patients*.

*^e^The distribution of genotype frequencies differed significantly between GD with and without GO patients*.

## Discussion

In this study, we replicated the association of the G allele of the rs7679475 (A/G) SNP within the *GPR103* gene with HT in Japanese population. It should be noted that a locus on chromosome 4q27 that is approximately 625 kB downstream the *GPR103* gene was previously reported to be associated with T1D (17), as well as with other autoimmune diseases, including rheumatoid arthritis (18), Celiac disease (19), and ulcerative colitis (20). However, there was no association between rs7679475 and GD. Tomer et al. (11) previously tested the two *GPR103* SNPs (rs1513695, and rs7679475) showing association with APS3v, for association with AITD in their AITD dataset. The analysis showed that the two SNPs were both associated with HT, but not with GD (11). Our data and the data of Tomer et al. suggested that the *GPR103* gene may contribute to the pathogenesis of HT in Caucasian and Japanese patients. The *GPR103* gene (also designated as *QRFPR* gene) is a G-protein-coupled receptor that is expressed not only in brain but also in human pancreatic islets, and it may have an effect on insulin secretion (21). The ligand for *GPR103* (*P518*) is expressed in thyroid cells (22). Therefore, the mechanism by which the *GPR103* gene may predispose to thyroid autoimmunity is still unclear.

The frequency of the G allele of the rs2358994 (A/G) was also found to be associated with GO in Japanese. The GWAS in patients with APS3v (11) mapped four genes (*BCL2L15, MAGI3, PHTF1*, and *PTPN22*) that are located in the same locus on chromosome 1p13 and are in linkage disequilibrium (LD) (11). Since the rs2476601 SNP within the *PTPN22* gene revealed no polymorphism in the Japanese (15), we tested three additional SNPs in this locus (rs2358994, rs2153977, and rs1111695) for association with AITD. Although both GD and HT showed no significant associations with any of these SNPs, there was a significant association between GO and rs2358994, suggesting that the *BCL2L15* gene may be a GO-specific gene in Japanese. SNP rs2358994 showed significant association only with GO, but not with GD without GO. This SNP is located within the *BCL2L15* gene, which is in tight LD with *PTPN22* (11). Therefore, at present, we cannot determine if this association reflects an association with the *BCL2L15* gene, *PTPN22* gene, or another gene within this locus, and further studies are needed to determine that.

The *BCL2L15* gene (also designated as BFK gene) is a proapoptotic member of the BCL2 family (23, 24). Reduction in *BCL2L15* protein expression is observed in gastrointestinal tumors (25). Interestingly, higher levels of the *BCL2* protein was registered in freshly isolated Crohn’s disease patient polymorphonuclear neutrophils, in contrast to controls, in which *BCL2* protein was undetectable (26). These data suggest that the defective functionality of neutrophils can be the early event responsible for the altered mucosal immune response in Crohn’s disease, which is one of the autoimmune diseases.

There are two limitations of the present study. First, the number of samples is relatively small that may have limited our ability to identify common variants with weak effects. Second, controls selection process may be insufficient. However, all the controls have been screened for T3/T4 of other thyroid-related phenotypes. Also, all study participants lived in the Tokyo metropolitan area. Moreover, quality control (QC) testing, including testing for HWE, was performed using SNPALyze ver. 7.0 (http://www.dynacom.co.jp/e/products/package/snpalyze/). Tests in subjects and controls did not show any significant deviation from HWE for any of the SNPs.

In conclusion, we replicated an association between HT and the G allele of rs7679475 within the *GPR103* gene in Japanese. These results suggest the *GPR103* gene may contribute to the pathogenesis of HT in both Caucasians and Japanese. We also, for the first time, identified the association of SNP rs2358994 within *BCL2L15* gene with GO in the Japanese population, suggesting genetic influences in different ethnic populations (27). Studies with ethnically diverse populations, including large numbers of patients, are needed to confirm the role of above two genes in AITD.

## Author Contributions

YB and TT: conceived, designed, and performed the experiments, analyzed the data, and contributed reagents/materials/analysis tools; YB, TT, and YN: wrote the paper.

## Conflict of Interest Statement

The authors declare that the research was conducted in the absence of any commercial or financial relationships that could be construed as a potential conflict of interest.
